# *EGFR* mutations in sinonasal squamous tumors: oncogenic and therapeutic implications

**DOI:** 10.18632/oncoscience.268

**Published:** 2015-11-19

**Authors:** Aaron M. Udager, Jonathan B. McHugh, Kojo S.J. Elenitoba-Johnson, Noah A. Brown

**Affiliations:** Department of Pathology, University of Michigan, Ann Arbor, MI, USA

**Keywords:** sinonasal, squamous, papilloma, EGFR, HPV

Sinonasal squamous cell carcinoma (SNSCC) and sinonasal papillomas constitute a diverse group of epithelial tumors arising in the sinonasal tract [1]. Sinonasal papillomas are benign tumors classified into three distinct histologic types: exophytic (fungiform), inverted, and oncocytic. While exophytic sinonasal papillomas (ESP) arise from the nasal septum and are only rarely associated with SNSCC, inverted sinonasal papillomas (ISP) and oncocytic sinonasal papillomas (OSP) typically arise from the lateral portion of the nasal cavity and are more frequently associated with synchronous or metachronous SNSCC – up to 25%, depending on the study [2].

The etiology of sinonasal tumors is a topic of current debate [1]. While ESP is associated with infection by low-risk human papillomavirus (HPV) in 55% - 65% [1, 3], most studies have demonstrated significantly lower HPV detections rates for ISP [3, 4]. Similarly, less than half of SNSCC are associated with HPV infection [3, 4] and the incidence in SNSCC associated with ISP may be even lower [5]. These data suggest that while HPV infection may play a role in the pathogenesis of a subset of these tumors, it is not the only factor involved in SNP and SNSCC oncogenesis.

In a recent study, our group identified activating somatic *EGFR* mutations in 88% of ISP and 77% of SNSCC associated with ISP [6]. Importantly, while a variety of different *EGFR* mutations were found in these tumors, concordant *EGFR* genotypes were identified for all matched pairs of ISP and synchronous or metachronous SNSCC. Therefore, this study provided the first molecular evidence to support the role of ISP as a precursor lesion for SNSCC. In addition, *EGFR* mutation status was a significant prognostic factor for ISP, with *EGFR* wild-type tumors showing earlier progression to SNSCC. No *EGFR* mutations were identified in ESP, OSP, or SNSCC not associated with ISP, suggesting that the ISP/SNSCC disease spectrum is biologically distinct from these other sinonasal squamous tumors.

**Figure 1:**
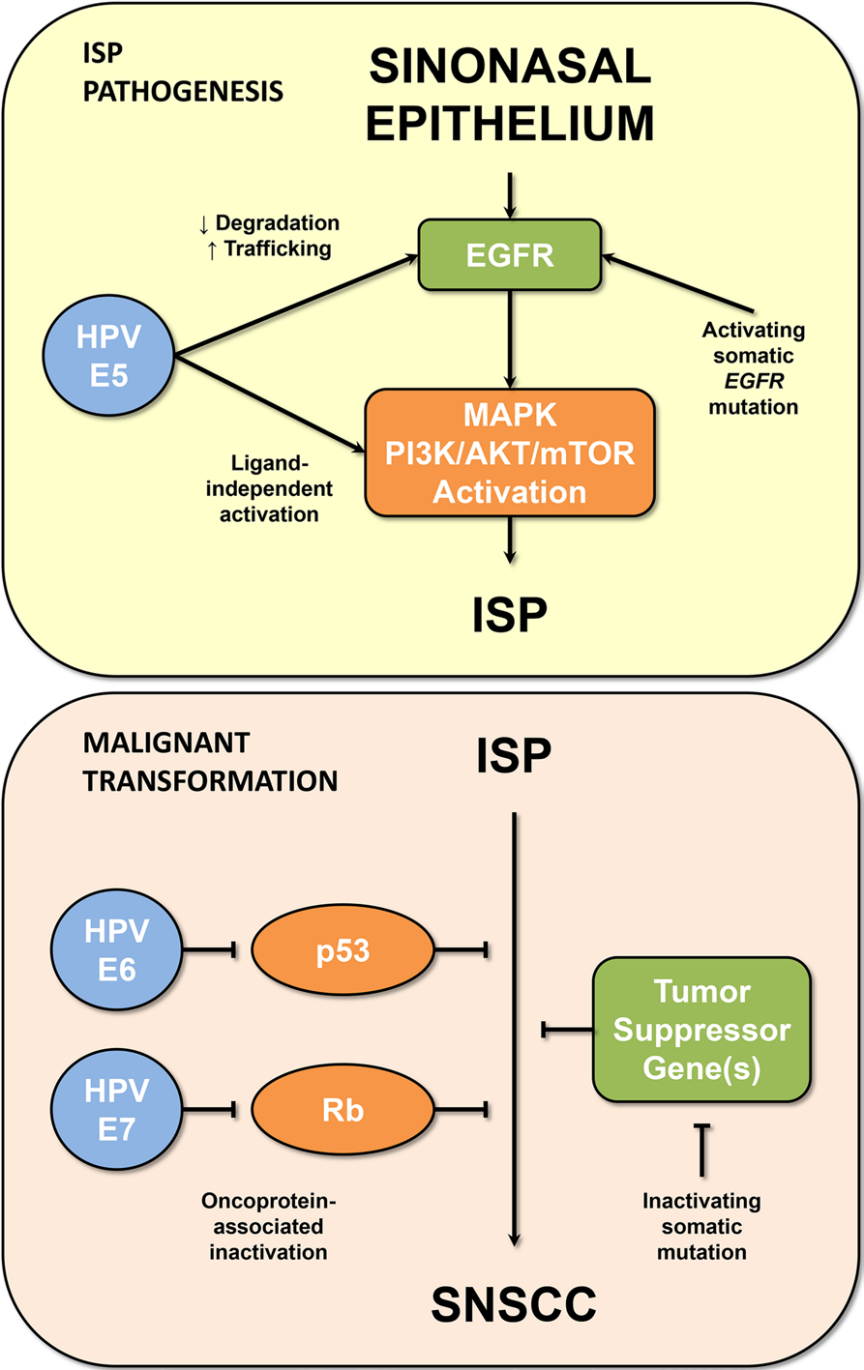
Possible mechanisms of ISP oncogenesis and malignant transformation (Upper panel) Activating somatic *EGFR* mutations have been shown to play a central role in the oncogenesis of 88% of ISP; however, HPV infection offers another possible mechanism of EGFR pathway activation (via the E5 oncoprotein). (Lower panel) Similarly, escape from oncogene-induced senescence and subsequent malignant transformation of ISP to SNSCC may involve inactivation of tumor suppressor proteins (i.e., p53 and Rb) through somatic mutation or HPV-associated E6 and E7 oncoproteins.

The oncogenic role of EGFR mutations was supported in this study by functional experiments. In cell lines derived from ISP-associated SNSCC, *EGFR* mutations were shown to result in activation of EGFR as well as downstream constituents of the MAPK and PI3K/AKT/mTOR signaling pathways. Taken together, these functional studies and the high frequency of *EGFR* mutations suggest that dysregulated EGFR signaling plays a central role in the oncogenesis of ISP and associated SNSCC – a finding that is an apparent departure from prior paradigms involving HPV infection [1]. These findings, however, do not strictly preclude a role for HPV in these tumors. The HPV-associated E5 oncoprotein has been shown to inhibit EGFR degradation, alter endosomal trafficking of EGFR and activate proteins downstream of EGFR in a ligand-independent manner [7] (Figure 1). Thus, it is plausible that altered EGFR signaling itself – either as a result of somatic activating *EGFR* mutations or HPV-associated E5 oncoprotein – is important in the development and evolution of ISP.

While there is now clear evidence to support the role of ISP as a precursor for SNSCC, the mechanism of progression remains uncharacterized. Progression of benign tumors is often restricted by oncogene-induced senescence, while escape from senescence and malignant transformation is often associated with loss of function of tumor suppressor proteins such as p53 or Rb. Inactivation of these tumor suppressors in cancer is frequently the result of somatic mutation; however, abrogation of p53 and Rb function by HPV-associated E6 and E7 proteins is another potential mechanism [1] (Figure 1). Future studies are needed both to correlate *EGFR* mutation and HPV infection status and to elucidate the mechanism of progression from ISP to SNSCC.

Surgical resection and radiotherapy is the current treatment of choice for SNSCC with chemotherapy generally reserved for locally advanced or metastatic SNSCC [8]. However, with these treatment options, SNSCC is associated with a 40% 5-year mortality. The discovery of *EGFR* mutations in ISP and associated SNSCC may provide an opportunity for the first targeted therapy in the treatment of these tumors. EGFR inhibitor therapy is now the standard of care in the treatment of advanced lung cancers with *EGFR* mutations. In contrast to lung cancers with *EGFR* exon 19 deletions, those with exon 20 insertions are generally resistant to the currently available reversible EGFR inhibitors gefitinib and erlotinib. However, irreversible EGFR inhibitors have been shown to have a more robust *in vitro* effect on exon 20 mutated lung cancer and clinical trials exploring their therapeutic potential are ongoing. Since the vast majority of *EGFR* mutations observed in ISP and associated SNSCC were exon 20 insertions, we explored the *in vitro* efficacy of both reversible and irreversible EGFR inhibitors [6]. As expected, ISP-associated SNSCC cells were relatively resistant to reversible EGFR inhibitors, while irreversible inhibitors resulted in robust growth inhibition as well as abrogation of EGFR, MAPK and AKT signaling. Further studies are now needed to determine the clinical utility of irreversible EGFR inhibitors in the treatment of ISP and associated SNSCC.
